# The combined effect of cigarette smoking and occupational noise exposure on hearing loss: evidence from the Dongfeng-Tongji Cohort Study

**DOI:** 10.1038/s41598-017-11556-8

**Published:** 2017-09-11

**Authors:** Dongming Wang, Zhichao Wang, Min Zhou, Wenzhen Li, Meian He, Xiaomin Zhang, Huan Guo, Jing Yuan, Yue Zhan, Kun Zhang, Tao Zhou, Weijia Kong, Weihong Chen

**Affiliations:** 1 Department of Occupational & Environmental Health, School of Public Health, Tongji Medical College, Huazhong University of Science and Technology, Wuhan, Hubei 430030 China; 2 Key Laboratory of Environment and Health, Ministry of Education & Ministry of Environmental Protection, and State Key Laboratory of Environmental Health (Incubating), School of Public Health, Tongji Medical College, Huazhong University of Science and Technology, Wuhan, Hubei 430030 China; 3 Wuhan Prevention and Treatment Center for Occupational Diseases, Wuhan, Hubei 430015 China; 4 Department of Otorhinolaryngology, Union Hospital, Tongji Medical College, Huazhong University of Science and Technology, Wuhan, Hubei 430030 China; 5 Department of Social Medicine and Health Management, School of Public Health, Tongji Medical College, Huazhong University of Science and Technology, Wuhan, Hubei 430030 China

## Abstract

Combined effect of cigarette smoking and occupational noise exposure on hearing loss has rarely been evaluated among Chinese population, especially among females. This cross-sectional study was conducted in 11196 participants of Dongfeng-Tongji cohort study. Smoking status was self-reported through questionnaire and occupational noise exposure was evaluated through workplace noise level and/or the job titles. Hearing loss was defined as a pure-tone mean of 25 dB or higher at 0.5, 1, 2, and 4 kHz in both ears. Compared with participants without occupational noise exposure, the risk of hearing loss was significantly higher for noise exposure duration ≥20 (OR = 1.45, 95%CI = 1.28–1.65). The association was particularly evident among individuals who were males (OR = 1.74, 95%CI = 1.45–2.08) and aged ≥ 70 (OR = 1.74, 95%CI = 1.30–2.33). Similarly, the risks increased with the increasing of pack-years in males and all age groups except for those aged <60. As to the combined effect, the hearing loss risk was highest for noise exposure duration ≥20 and pack-years ≥25 (OR = 2.41, 95%CI = 1.78–3.28), especially among males (OR = 2.42, 95%CI = 1.74–3.37) and those aged ≥70 (OR = 2.76, 95%CI = 1.36–5.60). Smoking may be an independent risk factor for hearing loss. And it may synergistically affect hearing when combined with occupational noise exposure, especially among males and older participants.

## Introduction

Occupational noise is considered one of the most pervasive occupational hazards around the world^1^. It is estimated that approximately 600 million workers worldwide and 30 million American workers are exposed to occupational noise^2, 3^. In Europe, it is reported that almost 28% of workers work in occupational settings with noise levels between 85 and 90 A-weighted decibels (dBA)^4^. And it may be more serious in China owing to its larger occupational population^5^.

Adverse health effects with exposure to occupational noise are of increasing health concern. Hearing loss is a well-known consequence of long-term exposure to high level noise^6, 7^, and occupational noise exposure has also been associated with sleeping disorders^8^, ECG abnormalities^9^ or hypertension^10^. Persistent damage on hair cells and hearing conduction nerves induced by long-term exposure to relative high level of occupational noise was thought to be a key reason for hearing loss^11^. Noise induced hearing loss (NIHL) is the change in the hearing threshold at different frequencies, it is chronic and characterized as sensorineural, usually symmetrical and bilateral^12^. NIHL could bring a lot of inconvenience to people in later life and cause a large social and economic burden, and hearing loss due to occupational noise has been described as a primary condition of modern society^13^. Published papers reported that an estimation of 278 million people were hearing disabilities in the world^14^, and the economic costs have been estimated to be about billions of dollars^15^. Although NIHL is permanent and irreversible, it is still preventable ^16^.

Except for occupational noise mentioned above, several other risk factors are also reported to be associated with hearing loss, such as age, sex, race, ototoxic medication, hypertension, and diabetes mellitus^17, 18^. Besides, cigarette smoking could also lead to elevated susceptibility to noise damage, as well as causing its own damage on hearing system^19^. Cigarette smoking is a common habit in the world, according to the World Health Organization, China has about 320 million smokers which represent one third of the world’s total smokers^20^. As a modifiable lifestyle, the association between cigarette smoking and hearing loss has been paid more attention in recent years. However, the results of the published reports were inconsistent.

Several epidemiology studies have found a positive association between smoking and hearing loss^21–23^, but others do not support a uniform relationship^24, 25^. The association between smoking and hearing loss has rarely been evaluated among female participants and evidence is limited in China. Moreover, the combined effect involving smoking and occupational noise exposure on hearing loss is noticeable due to the high prevalence of smoking among occupational workers in China^26^. Therefore, we conducted a cross-sectional study to examine the independent and combined effects of smoking and occupational noise exposure on hearing loss in a large middle-aged and older Chinese population, especially to explore the association among female participants.

## Results

### Descriptive

Characteristics of the 11196 participants included in the analysis were reported by categories of hearing loss (Table 1). Among them, 54.8% of participants were females, and 84.1% aged over 60. Overall, 34.7% were exposed to occupational noise (38.0% for male, 1922/5060; 32.1% for female, 1969/6136) and 14.7% were current smokers (37.2% for male, 1883/5060; 2.9% for female, 180/6136). The number of pack-years of smoking ranged from 0 to 195, with an average of 34.6 pack-years for current smokers and 24.9 for ex-smokers. The prevalence of hearing loss among all participants was 61.5% (72.9% for male and 52.1% for female). We observed pronounced differences in hearing loss prevalence by demographic characteristics. Prevalence of hearing loss was higher among men, aged over 70, current drinkers and subjects with hypertension, diabetes mellitus, coronary heart disease, myocardial infarction, and stroke. Current smokers and those exposed to occupational noise for 20 years or more were more inclined to have hearing loss.

**Table 1:** Characteristics of participants by hearing loss categories.

**Characteristics**	Total (N = 11196)	Hearing loss (N = 6888)	No hearing loss (N = 4308)	*P* value
**Age (year, mean ± SD)**	67.09 ± 7.38	69.31 ± 6.93	63.55 ± 6.67	<0.001
**Age**				<0.001
<60	1780(15.9)	538(7.8)	1242(28.8)	
60 ~ <70	5505(49.2)	3189(46.3)	2316(53.8)	
≥70	3911(34.9)	3161(45.9)	750(17.4)	
Sex				<0.001
Male	5060(45.2)	3691(53.6)	1369(31.8)	
Female	6136(54.8)	3197(46.4)	2939(68.2)	
Race				0.024
Han	11025(98.5)	6797(98.7)	4228(98.1)	
Others	171(1.5)	91(1.3)	80(1.9)	
**Shift work**				0.301
No	6184(55.2)	3831(55.6)	2353(54.6)	
Yes	5012(44.8)	3057(44.4)	1955(45.4)	
**Drinking status**				<0.001
Nondrinkers	7837(70.0)	4628(67.2)	3209(74.5)	
Ex-drinkers	743(6.6)	546(7.9)	197(4.6)	
Current drinkers	2616(23.4)	1714(24.9)	902(20.9)	
**Hypertension**				<0.001
No	3457(30.9)	1856(27.0)	1601(37.2)	
Yes	7739(69.1)	5032(73.0)	2707(62.8)	
**Diabetes mellitus**				0.001
No	9437(84.3)	5743(83.4)	3694(85.8)	
Yes	1759(15.7)	1145(16.6)	614(14.2)	
**Coronary heart disease**			<0.001	
No	9166(81.9)	5469(79.4)	3697(85.8)	
Yes	2030(18.1)	1419(20.6)	611(14.2)	
**Myocardial infarction**				<0.001
No	10800(96.5)	6582(95.6)	4218(97.9)	
Yes	396(3.5)	306(4.4)	90(2.1)	
Stroke				<0.001
No	10667(95.3)	6497(94.3)	4170(96.8)	
Yes	529(4.7)	391(5.7)	138(3.2)	
**Ototoxicity medicine**				0.55
No	8426(75.3)	5197(75.5)	3229(75.0)	
Yes	2770(24.7)	1691(24.5)	1079(25.0)	
**Occupational noise exposure**			<0.001	
No	7305(65.3)	4463(64.8)	2842(66.0)	
1≤ year <10	944(8.4)	507(7.4)	437(10.1)	
10≤ year <20	1150(10.3)	685(9.9)	465(10.8)	
year ≥ 20	1797(16.0)	1233(17.9)	564(13.1)	
**Smoking status**				<0.001
Nonsmokers	7484(66.9)	4171(60.6)	3313(76.9)	
Ex-smokers	2063(18.4)	1481(21.5)	582(13.5)	
Current smokers	1649(14.7)	1236(17.9)	413(9.6)	
Pack-years of smoking*			<0.001	
0	7484(69.9)	4171(63.8)	3313(79.6)	
0 ~ <25	1501(14.0)	1083(16.5)	418(10.1)	
≥25	1717(16.1)	1288(19.7)	429(10.3)	

^*^A total of 494 cases for missing data, and 346 for hearing loss.

### Occupational noise exposure and hearing loss

Table 2 presented the odds ratios (ORs) and 95% confidence intervals (CIs) for the effect of occupational noise exposure on hearing loss. Compared with participants not exposed to occupational noise, the risk of hearing loss was significantly higher among noise exposure duration for 20 years or more group (OR = 1.45, 95%CI = 1.28–1.65), after adjusting for potential confounders. Stratified analyses revealed that the association between the longest noise exposure duration group (≥20 years) and hearing loss was significant in different sex and age group except for those aged less than 60. And they were more pronounced in males (OR = 1.74, 95%CI = 1.45–2.08) and those aged over 70 (OR = 1.74, 95%CI = 1.30–2.33). Meanwhile, the association was also found among females (OR = 1.21, 95%CI = 1.01–1.44) and those aged 60 ~ <70 (OR = 1.37, 95%CI = 1.17–1.61).

**Table 2:** Odds ratios (95% CIs) of hearing loss by occupational noise exposure.

**Occupational noise exposure**	**N**	**Model 1** ^*^	**Model 2** ^†^	**Model 3** ^‡^
**Total**				
No	7305	ref	ref	ref
1 ≤ year <10	944	0.74(0.64–0.85)	0.94(0.81–1.10)	0.93(0.80–1.09)
10 ≤ year <20	1150	0.94(0.82–1.06)	1.15(0.99–1.33)	1.14(0.99–1.32)
year ≥20	1797	**1.38(1.23–1.54)**	**1.50(1.33–1.69)**	**1.45(1.28–1.65)**
**Sex**				
**Male**				
No	3138	ref	ref	ref
1 ≤ year <10	382	0.78(0.63–0.98)	0.90(0.71–1.14)	0.88(0.69–1.14)
10 ≤ year <20	425	1.04(0.83–1.30)	1.21(0.95–1.54)	1.13(0.88–1.45)
year ≥20	1115	**1.59(1.34–1.87)**	**1.78(1.50–2.11)**	**1.74(1.45–2.08)**
**Female**				
No	4167	ref	ref	ref
1 ≤ year <10	562	0.72(0.60–0.86)	0.97(0.80–1.18)	0.96(0.79–1.16)
10 ≤ year <20	725	0.95(0.81–1.11)	1.15(0.97–1.37)	1.14(0.95–1.35)
year ≥20	682	0.87(0.74–1.02)	**1.25(1.05–1.49)**	**1.21(1.01–1.44)**
**Age Group, y**				
<60				
No	1048	ref	ref	ref
1 ≤ year <10	221	1.04(0.76–1.43)	1.04(0.76–1.42)	1.00(0.72–1.38)
10 ≤ year <20	254	0.91(0.67–1.24)	0.90(0.66–1.22)	0.90(0.66–1.22)
year ≥20	257	1.18(0.88–1.58)	1.18(0.88–1.58)	1.10(0.82–1.49)
60 ~ <70				
No	3491	ref	ref	ref
1 ≤ year <10	480	0.76(0.63–0.92)	0.76(0.62–0.92)	0.75(0.62–0.92)
10 ≤ year <20	527	1.13(0.94–1.37)	1.18(0.98–1.43)	1.18(0.98–1.44)
year ≥20	1007	**1.63(1.40–1.89)**	**1.40(1.20–1.62)**	**1.37(1.17–1.61)**
** ≥70**				
No	2766	ref	ref	ref
1 ≤ year <10	243	1.29(0.91–1.83)	1.28(0.90–1.81)	1.26(0.87–1.81)
10 ≤ year <20	369	1.18(0.89–1.56)	1.18(0.90–1.57)	1.11(0.83–1.47)
year ≥20	533	**1.79(1.36–2.35)**	**1.73(1.31–2.27)**	**1.74(1.30–2.33)**

^*^Unadjusted.

^†^Adjusted for age/sex.

^‡^Adjusted for age/sex, race, shift work, smoking status, drinking status, hypertension, ototoxicity medicine, chronic diseases (diabetes mellitus, coronary heart disease, myocardial infarction and stroke).

### Smoking and hearing loss

The effect of smoking status on hearing loss was revealed in Table S1. Compared with nonsmokers, current smokers had higher risk of hearing loss (OR = 1.38, 95%CI = 1.20–1.59). Stratified analyses indicated that the association was significant in males (OR = 1.34, 95%CI = 1.14–1.58) and all age groups but the oldest age group (≥70). Besides, it was not significant in females either (OR = 1.29, 95%CI = 0.92–1.82).

In order to explore whether the risk varied by amount of smoking exposure, the association between pack-years of exposure and hearing loss was also further evaluated in Table 3. The odds ratios increased with the increasing of pack-years, and the highest exposure category (≥25 pack-years) got the highest risk (OR = 1.42, 95%CI = 1.21–1.66). In the stratified analysis, the similar trend was also found in males, and all age groups except for the youngest age group (<60). Besides, the association between pack-years of smoking and hearing loss was also found among females, the risk of hearing loss was statistically significant (OR = 1.54, 95%CI = 1.02–2.33) in the median exposure category (0 ~ <25 pack-years), not the highest exposure group (OR = 1.42, 95%CI = 0.71–2.84).

**Table 3:** Odds ratios (95% CIs) of hearing loss by pack-years of smoking.

**Pack-years of smoking** ^**#**^	**N**	Model 1*	**Model 2** ^†^	**Model 3** ^‡^
**Total**				
0	7484	ref	ref	ref
0 ~ <25	1501	**2.05(1.82–2.32)**	**1.33(1.14–1.55)**	**1.29(1.10–1.51)**
≥25	1717	**2.36(2.10–2.66)**	**1.46(1.25–1.70)**	**1.42(1.21–1.66)**
**Sex**				
**Male**				
0	1600	ref	ref	ref
0 ~ <25	1372	1.04(0.89–1.22)	**1.25(1.06–1.48)**	**1.22(1.02–1.45)**
≥25	1666	**1.18(1.01–1.38)**	**1.40(1.19–1.65)**	**1.37(1.16–1.62)**
**Female**				
0	5884	ref	ref	ref
0 ~ <25	129	**2.10(1.44–3.06)**	**1.57(1.04–2.36)**	**1.54(1.02–2.33)**
≥25	51	**2.68(1.42–5.05)**	1.43(0.71–2.88)	1.42(0.71–2.84)
**Age Group, y**				
** <60**				
0	1610	ref	ref	ref
0~ <25	88	**1.94(1.26–2.99)**	**2.11(1.20–3.70)**	**2.00(1.13–3.54)**
≥25	58	1.38(0.80–2.38)	1.54(0.76–3.11)	1.48(0.72–3.05)
**60 ~ <** **70**				
0	3517	ref	ref	ref
0 ~ <25	798	**1.84(1.56–2.16)**	1.15(0.94–1.41)	1.15(0.93–1.42)
≥25	946	**2.07(1.78–2.42)**	**1.26(1.03–1.54)**	**1.26(1.03–1.55)**
** ≥70**				
0	2357	ref	ref	ref
0~ <25	615	**1.30(1.03–1.63)**	1.27(0.99–1.64)	1.22(0.94–1.58)
≥25	713	**1.53(1.22–1.93)**	**1.51(1.17–1.94)**	**1.45(1.11–1.88)**

^*^Unadjusted.

^†^Adjusted for age/sex.

^‡^Adjusted for age/sex, race, shift work, occupational noise exposure, drinking status, hypertension, ototoxicity medicine, chronic diseases (diabetes mellitus, coronary heart disease, myocardial infarction and stroke).

^#^The sample sizes vary slightly because of missing data.

### Combined association of occupational noise exposure and smoking on hearing loss

We further explored the combined association of occupational noise exposure and smoking on hearing loss (Table S2). Compared with no-smoking and no occupational noise exposed participants, all other groups had higher risks of hearing loss except for the ex-smokers and not exposed to occupational noise, and those exposed to occupational noise and smoking got the highest risk (OR = 1.96, 95%CI = 1.60–2.41). Meanwhile, the combined associations were also found in the subgroup analyses, especially among males (OR = 2.06, 95%CI = 1.63–2.59) and the youngest age group (OR = 2.74, 95%CI = 1.41–5.34).

As no occupational noise exposure and no smoking were treat as the reference group. The association of pack-years of smoking and noise exposure duration was further assessed to test the dose-response relationship (Table 4). Individuals with longer noise exposure duration (≥20 years) and more pack-years of smoking (≥25 pack-years) got the highest risk (OR = 2.41, 95%CI = 1.78–3.28). In the subgroup analyses, the combined associations were also found in males (OR = 2.42, 95%CI = 1.74–3.37), participants aged ranging from 60 to 70 (OR = 1.96, 95%CI = 1.36–2.81) and aged over 70 (OR = 2.76, 95%CI = 1.36–5.60), not in females (OR = 2.07, 95%CI = 0.17–25.41) and aged less than 60 (OR = 2.81, 95%CI = 0.74–10.67).

**Table 4:** Odds ratios (95% CIs) of hearing loss by combined categories of occupational noise exposure and pack-years of smoking.

Pack-years of smoking^#^	Occupational noise exposure							
	No		1 ≤ year <10		10 ≤ year <20		year ≥ 20	
	N	OR (95%CI)	N	OR (95%CI)	N	OR (95%CI)	N	OR (95%CI)
**Total** ^*^								
0	5021	ref	673	0.92(0.77–1.09)	835	1.17(0.99–1.37)	955	**1.32(1.13–1.54)**
0 ~ <25	945	**1.24(1.03–1.49)**	106	0.91(0.59–1.41)	127	1.25(0.82–1.92)	323	**2.13(1.62–2.79)**
≥25	1021	**1.36(1.13–1.63)**	125	**1.71(1.10–2.67)**	155	1.43(0.96–2.11)	416	**2.41(1.78–3.28)**
**Sex** ^†^								
**Male**								
0	1029	ref	126	0.78(0.52–1.17)	135	1.35(0.87–2.08)	310	**1.79(1.30–2.45)**
0 ~ <25	861	1.23(0.99–1.52)	95	0.87(0.55–1.38)	111	1.26(0.80–2.00)	305	**2.15(1.61–2.86)**
≥25	978	**1.39(1.13–1.71)**	125	**1.71(1.09–2.68)**	151	1.44(0.96–2.15)	412	**2.42(1.74–3.37)**
**Female**								
0	3992	ref	547	0.96(0.79–1.17)	700	1.15(0.96–1.37)	645	**1.20(1.00–1.44)**
0~ <25	84	1.60(0.97–2.65)	11	1.55(0.33–7.24)	16	1.13(0.34–3.71)	18	2.07(0.70–6.11)
≥25	43	1.47(0.69–3.13)	—	—	4	0.86(0.08–9.30)	4	2.07(0.17–25.41)
**Age Group, y** ^‡^								
** <60**								
0	959	ref	197	1.03(0.73–1.44)	228	0.87(0.62–1.20)	226	1.03(0.75–1.42)
0 ~ <25	48	**2.38(1.21–4.68)**	14	0.39(0.08–1.92)	12	2.41(0.67–8.62)	14	3.06(0.99–9.50)
≥25	28	0.67(0.24–1.89)	8	**4.70(1.19–17.35)**	12	1.38(0.37–5.15)	10	2.81(0.74–10.67)
**60** ~ <**70**								
0	2328	ref	329	0.75(0.60–0.95)	378	**1.31(1.05–1.64)**	482	1.21(0.99–1.48)
0 ~ <25	482	1.15(0.90–1.47)	54	0.59(0.34–1.03)	55	0.82(0.47–1.45)	207	**1.86(1.34–2.58)**
≥25	524	1.19(0.93–1.52)	74	1.19(0.71–1.99)	82	1.16(0.71–1.90)	266	**1.96(1.36–2.81)**
≥**70**								
0	1734	ref	147	1.08(0.71–1.64)	229	1.08(0.77–1.52)	247	**1.68(1.15–2.45)**
0~ <25	415	1.08(0.81–1.45)	38	3.14(0.95–10.33)	60	1.57(0.76–3.26)	102	**2.16(1.24–3.74)**
≥25	469	**1.47(1.09–1.99)**	43	2.02(0.78–5.24)	61	1.41(0.70–2.84)	140	**2.76(1.36–5.60)**

^*^Adjusted for sex, age, race, shift work, drinking status, hypertension, ototoxicity medicine, chronic diseases (diabetes mellitus, coronary heart disease, myocardial infarction and stroke).

^†^Adjusted for age, race, shift work, smoking status, drinking status, hypertension, ototoxicity medicine, chronic diseases (diabetes mellitus, coronary heart disease, myocardial infarction and stroke).

^‡^Adjusted for sex, race, shift work, smoking status, drinking status, hypertension, ototoxicity medicine, chronic diseases (diabetes mellitus, coronary heart disease, myocardial infarction and stroke).

^#^The sample sizes vary slightly because of missing data.

## Discussion

To our best of knowledge, few studies evaluated the association of smoking and hearing loss among female subjects, as the low percentage of smokers among this group. And we also explored the independent and combined dose-response association of occupational noise exposure and smoking with hearing loss. Moreover, the data of our study were based on a large middle-aged and older Chinese population, which could provide more reliable results.

In the present study of middle-aged and older adults in Chinese retired workers, occupational noise exposure and cigarette smoking, independently or in combination, were found to be associated with increased risk of hearing loss. High risks of hearing loss were observed among participants long-term exposed to occupational noise and/or took more pack-years of smoking, even controlling for other confounders. Long-term exposed to occupational noise could damage hair cells of the organ of Corti directly, resulting in irreversible and progressive hearing loss^7^, which was known as noise-induced hearing loss (NIHL). Besides, it also revealed that smoking may be an independent risk factor for hearing loss and it may have a dose-response relationship on hearing loss, which was consistent with part of previous studies^27–29^. The divergent outcome may be due to the different definitions and criteria of NIHL among studies. Though the mechanism by which cigarette smoking increased the risk of hearing loss was not clear, it was indicated that smoking may damage hair cells by increasing carboxyhaemogolobin or by reducing blood flow to cochlea^23, 30, 31^.

Our study showed the combined effect of smoking and occupational noise exposure was greater than their independent effects. Individuals with longest noise exposure duration and maximum pack-years got the highest risk in the present study, which was reported in other studies. Pouryaghoub *et al*. in Iran found smoking could accelerate noise induced hearing loss^32^, Ferrite and Santana in Brazil found smoking may synergistically affect hearing when in combination with noise exposure^33^. Mizoue *et al*. in Japan also found smoking was a risk factor for high frequency hearing loss and had a positive combined association with occupational noise exposure^19^. However, these studies were conducted among male workers, not including female workers. The results of our study showed the prevalence of hearing loss in females was lower than that in male participants. However, increased risks of hearing loss were also observed among female participants with cigarette smoking and long term exposure to occupational noise. It was concluded that low percent of smoking (male vs female, 37.2% *vs* 2.9%) or exposure to occupational noise (male *vs* female, 38.0% vs 32.1%) may contribute to the phenomenon in females.

In the present study, we also explored the independent and combined associations in the subgroup of sex and age. During the independent association, we did not found significant associations between maximum pack-years of smoking (≥25 pack-years) and hearing loss among females and the youngest age group (<60). Meanwhile, the combined associations were not found in aforementioned two groups either. However, these associations could also be found in other female (0 ~ <25 pack-years) and age groups (60 ~ <70 and ≥70). It may mainly be due to the small number of participants in these categories (ranging from 4 to 58), which resulting a limited statistical power. Thus the independent and combined associations among females and the youngest age group (<60) needed to be confirmed further in future studies with larger sample size.

Some limitations in the present study should also be acknowledged. First, our study was a cross-sectional design, which would restrict the evidence of causal inferences. Second, although a number of confounders were adjusted in our study, there were still some other factors which were not included, such as leisure time noise exposure, which was also reported to be associated with hearing loss^17^. However, the sample size of the present study was large, which could reduce possible bias. Thirdly, the results of our study were limited to middle-aged and older adults, thus it may not be generalized to populations of all ages.

In summary, the present study reveals that smoking is an independent risk factor for hearing loss. And it may synergistically affect hearing when in combination with occupational noise exposure, especially among males and older participants. The associations among females and the youngest age group need to be confirmed in future studies with larger sample size.

## Methods

### Study population

The study is embedded in Dongfeng-Tongji Cohort Study, a population based cohort study aimed at assessing the relationship of dietary, lifestyle, occupational and environmental factors and the development of chronic diseases, which has been reported elsewhere^34^. Briefly, all the participants were retired employees from Dongfeng Motor Corporation (DMC), each participant has a unique medical insurance card number in the DMC’s health-care service system. The cohort study was established in 2008 and was followed up for every five years. Finally, a total of 27,009 retired employees completed baseline questionnaires and medical examinations. And five years later, 25,978 individuals (96.2%) completed the follow-up until October 2013. In 2013, 11513 participants from the baseline underwent the audiometric examination. Further excluded from this group were individuals without auditory examination results (n = 57), those who provided incomplete baseline information (n = 100), leaving 11196 subjects for the present analysis.

### Auditory measures and ascertainment of hearing loss

Each subject was given a general physical and an otologic examination first. Then pure-tone audiometry was performed in a sound-isolated room with a calibrated pure-tone audiometer (Micro-CD21) by certified audiologists. Air conduction thresholds were determined for each ear at 0.5, 1, 2, 4 and 8 kHz across an intensity range of −10 to 120 dB. The non-responses were coded as missing values. As occupational hearing loss was usually supposed to bilateral and fairly symmetrical^35^, it was defined as a pure-tone mean of 25 dB or higher at 0.5, 1, 2, and 4 kHz in both ears^36, 37^.

### Noise exposure assessment

The occupational information of each subject was self-reported and obtained from questionnaire, which contained personal data on employment and variables about the work, such as the corporation, the job title and the duration of each job. The occupational noise level for each job title at workplace came from the company records which were measured by qualified institutions. Noise exposure levels for workplaces outside of DMC were determined through the job description, and were consulted with local occupational hygienists. The occupational noise exposure was defined as exposed to a time-weighted-average (TWA) noise level of 80 dB (A) for at least a year^29^. Besides, years of occupational noise exposure was divided into four groups: 0, 1 ~ <10, 10 ~ <20, ≥20.

### Smoking assessment

Smoking status was self-reported and it was divided into three groups: current smokers, ex-smokers and nonsmokers. Individuals smoking at least one cigarette per day for more than half a year were defined as current smokers. Nonsmokers were defined as those who seldom or had never smoked in their lifetime. And ex-smokers were those who ever smoke and do not smoke at present. The number of cigarettes smoked or ever smoked per day and the duration of smoking was also recorded for current smokers and ex-smokers. Meanwhile, total pack-years were defined as the number of cigarettes smoked per day divided by 20 per pack, and then multiplied by years of smoking^21^.

### Ethical approval

The study was approved by the Ethics and Human Subject committee of Tongji Medical College, and Dongfeng General Hospital, DMC. The methods were carried out in accordance with the relevant guidelines and regulations. The written informed consents were obtained from all the participants.

### Covariates

Information on sociodemographic characteristics (sex, age, and race), shift work, drinking status, hypertension, ototoxicity medicine, and chronic diseases history were collected through a questionnaire by face to face interview with trained interviewers. As hearing loss varied by age^17^, it was classified into three groups: <60, 60 ~ <70, ≥70. Individuals who drink at least one time per week for more than half a year were defined as current drinkers. And non-drinkers were defined as those who seldom or had never drank in their lifetime. Hypertension was defined as blood pressure ≥140/90 mmHg or self-reported physician diagnosis of hypertension or self-reported current use of antihypertensive medication. Use of ototoxic medication was counted when participants reported medications of loop diuretics, aminoglycoside or non-steroidal anti-inflammatory drugs. Chronic diseases history diagnosed by a physician was reported by the participants, including diabetes mellitus, coronary heart disease, myocardial infarction, and stroke.

### Statistical Analysis

Sociodemographic characteristic of participants was reported as mean (SD) for continuous variables and as number (percentages) for categorical variables. Logistic regressions were performed to evaluate the independent and combined associations of occupational noise exposure and smoking with hearing loss. Then the associations were further evaluated with stratified sex and age, based on previous published reports suggesting that age and sex may be important factors for hearing loss^21, 38^. The models were conducted with those do not smoke and no occupational noise exposure as the reference group. We chose covariates which could affect hearing loss according to evidence from published literatures^17, 21^. Covariates included age, sex, race, shift work, drinking status, hypertension, ototoxicity medicine, and chronic diseases history (diabetes mellitus, coronary heart disease, myocardial infarction and stroke). All statistical analyses were performed using SAS version 9.2 software (SAS institute Inc., Cary, NC). The statistical tests were two sided, and significance was set at *P* < 0.05.

### Data Availability

The datasets generated during and/or analyzed during the current study are available from the corresponding author on reasonable request.

## Electronic supplementary material

Table S1, Table S2
