# Supplementary material for: The combined effect of cigarette smoking and occupational noise exposure on hearing loss: evidence from the Dongfeng-Tongji Cohort Study
Source: Sci Rep. 2017 Sep 11;7:11142. doi: 10.1038/s41598-017-11556-8 (PMC5593900; doi:10.1038/s41598-017-11556-8)
Supplement: Supplementary file 1 — Table S1, Table S2 [file 41598_2017_11556_MOESM1_ESM.doc]

**The combined effect of cigarette smoking and occupational noise exposure on hearing loss: evidence from the Dongfeng-Tongji Cohort Study**

Dongming Wang ^1,2,3^, Zhichao Wang ^4^, Min Zhou ^1,2^, Wenzhen Li ^5^, Meian He ^1,2^, Xiaomin Zhang ^1,2^, Huan Guo ^1,2^, Jing Yuan ^1,2^, Yue Zhan ^4^, Kun Zhang ^4^, Tao Zhou ^4^, Weijia Kong ^4^,*, Weihong Chen ^1,2^,*

**Table S1 Odds ratios (95% CIs) of hearing loss by smoking status**

| **Smoking status** | **N** | **Model 1**^*^ | **Model 2**^†^ | **Model 3**^‡^ |
| --- | --- | --- | --- | --- |
| **Total** |  |  |  |  |
| Nonsmokers | 7484 | ref | ref | ref |
| Current smokers | 2063 | **2.02(1.82-2.25)** | **1.42(1.24-1.64)** | **1.38(1.20-1.59)** |
| Ex-smokers | 1649 | **2.38(2.11-2.68)** | **1.26(1.08-1.47)** | **1.19(1.01-1.39)** |
| **Sex** |  |  |  |  |
| **Male** |  |  |  |  |
| Nonsmokers | 1600 | ref | ref | ref |
| Current smokers | 1883 | 1.05(0.90-1.21) | **1.39(1.19-1.63)** | **1.34(1.14-1.58)** |
| Ex-smokers | 1577 | **1.20(1.03-1.41)** | **1.19(1.01-1.39)** | 1.17(0.99-1.39) |
| **Female** |  |  |  |  |
| Nonsmokers | 5884 | ref | ref | ref |
| Current smokers | 180 | **1.80(1.32-2.45)** | 1.31(0.93-1.84) | 1.29(0.92-1.82) |
| Ex-smokers | 72 | **2.45(1.46-4.12)** | 1.58(0.89-2.82) | 1.48(0.83-2.64) |
| **Age Group, y** |  |  |  |  |
| **<60** |  |  |  |  |
| Nonsmokers | 1610 | ref | ref | ref |
| Current smokers | 133 | **1.72(1.20-2.46)** | **1.92(1.16-3.18)** | **1.86(1.11-3.11)** |
| Ex-smokers | 37 | 1.48(0.76-2.90) | 1.61(0.78-3.34) | 1.53(0.72-3.21) |
| **60~<70** |  |  |  |  |
| Nonsmokers | 3517 | ref | ref | ref |
| Current smokers | 1213 | **1.96(1.71-2.25)** | **1.21(1.01-1.46)** | **1.21(1.00-1.47)** |
| Ex-smokers | 775 | **1.77(1.51-2.09)** | 1.07(0.87-1.32) | 1.06(0.86-1.32) |
| **≥70** |  |  |  |  |
| Nonsmokers | 2357 | ref | ref | ref |
| Current smokers | 717 | **1.33(1.07-1.66)** | **1.32(1.04-1.67)** | 1.25(0.98-1.60) |
| Ex-smokers | 837 | **1.50(1.21-1.85)** | **1.48(1.16-1.88)** | **1.32(1.03-1.69)** |

^*^Unadjusted.

^†^Adjusted for age/sex.

^‡^Adjusted for age/sex, race, shift work, occupational noise exposure, drinking status, hypertension, ototoxicity medicine, chronic diseases (diabetes mellitus, coronary heart disease, myocardial infarction and stroke).

**Table S2 Odds ratios (95% CIs) of hearing loss by combined categories of occupational noise exposure and smoking status**

| **Variables** |  | **Occupational noise exposure** | | | |
| --- | --- | --- | --- | --- | --- |
|  | **No** | |  | **Yes** | |
|  | **N** | **OR (95%CI)** |  | **N** | **OR (95%CI)** |
| **Total**^*^ |  |  |  |  |  |
| Nonsmokers | 5021 | ref |  | 2463 | **1.15(1.03-1.28)** |
| Ex-smokers | 1018 | 1.18(0.98-1.42) |  | 631 | **1.43(1.14-1.78)** |
| Current smokers | 1266 | **1.26(1.07-1.48)** |  | 797 | **1.96(1.60-2.41)** |
| **Sex**^†^ |  |  |  |  |  |
| **Male** |  |  |  |  |  |
| Nonsmokers | 1029 | ref |  | 571 | **1.38(1.08-1.76)** |
| Ex-smokers | 973 | **1.23(1.00-1.52)** |  | 604 | **1.50(1.18-1.91)** |
| Current smokers | 1136 | **1.30(1.07-1.58)** |  | 747 | **2.06(1.63-2.59)** |
| **Female** |  |  |  |  |  |
| Nonsmokers | 3992 | ref |  | 1892 | 1.11(0.98-1.25) |
| Ex-smokers | 45 | 1.77(0.83-3.77) |  | 27 | 1.29(0.52-3.20) |
| Current smokers | 130 | 1.29(0.87-1.92) |  | 50 | 1.49(0.77-2.89) |
| **Age Group, y**^‡^ |  |  |  |  |  |
| **<60** |  |  |  |  |  |
| Nonsmokers | 959 | ref |  | 651 | 0.97(0.78-1.21) |
| Ex-smokers | 17 | 2.19(0.80-6.03) |  | 20 | 1.15(0.41-3.23) |
| Current smokers | 72 | 1.39(0.75-2.57) |  | 61 | **2.74(1.41-5.34)** |
| **60**~<**70** |  |  |  |  |  |
| Nonsmokers | 2328 | ref |  | 1189 | 1.09(0.94-1.26) |
| Ex-smokers | 447 | 1.09(0.85-1.41) |  | 328 | 1.13(0.85-1.50) |
| Current smokers | 716 | 1.12(0.90-1.40) |  | 497 | **1.57(1.22-2.02)** |
| **≥70** |  |  |  |  |  |
| Nonsmokers | 1734 | ref |  | 623 | **1.27(1.00-1.60)** |
| Ex-smokers | 554 | 1.22(0.93-1.62) |  | 283 | **2.08(1.38-3.15)** |
| Current smokers | 478 | 1.17(0.89-1.55) |  | 239 | **1.94(1.28-2.96)** |

^*^Adjusted for sex, age, race, shift work, drinking status, hypertension, ototoxicity medicine, chronic diseases (diabetes mellitus, coronary heart disease, myocardial infarction and stroke).

^†^Adjusted for age, race, shift work, smoking status, drinking status, hypertension, ototoxicity medicine, chronic diseases (diabetes mellitus, coronary heart disease, myocardial infarction and stroke).

^‡^Adjusted for sex, race, shift work, smoking status, drinking status, hypertension, ototoxicity medicine, chronic diseases (diabetes mellitus, coronary heart disease, myocardial infarction and stroke).
